# Zebrafish Mutant Lines Reveal the Interplay between *nr3c1* and *nr3c2* in the GC-Dependent Regulation of Gene Transcription

**DOI:** 10.3390/ijms23052678

**Published:** 2022-02-28

**Authors:** Alberto Dinarello, Annachiara Tesoriere, Paolo Martini, Camilla Maria Fontana, Davide Volpato, Lorenzo Badenetti, Francesca Terrin, Nicola Facchinello, Chiara Romualdi, Oliana Carnevali, Luisa Dalla Valle, Francesco Argenton

**Affiliations:** 1 Department of Biology, University of Padova, 35121 Padova, Italy; alberto.dinarello@phd.unipd.it (A.D.); annachiara.tesoriere@phd.unipd.it (A.T.); camillamaria.fontana@phd.unipd.it (C.M.F.); davide.volpato.4@studenti.unipd.it (D.V.); lorenzo.badenetti@phd.unipd.it (L.B.); francesca.terrin@phd.unipd.it (F.T.); nicola.facchinello@unipd.it (N.F.); chiara.romualdi@unipd.it (C.R.); francesco.argenton@unipd.it (F.A.); 2 Department of Molecular and Translational Medicine, University of Brescia, 25121 Brescia, Italy; paolo.martini@unibs.it; 3 Department of Life and Environmental Sciences, Università Politecnica delle Marche, 60131 Ancona, Italy; o.carnevali@univpm.it

**Keywords:** glucocorticoid receptor, mineralocorticoid receptor, zebrafish, CRISPR/Cas9

## Abstract

Glucocorticoids mainly exert their biological functions through their cognate receptor, encoded by the *nr3c1* gene. Here, we analysed the glucocorticoids mechanism of action taking advantage of the availability of different zebrafish mutant lines for their receptor. The differences in gene expression patterns between the zebrafish *gr* knock-out and the *gr^s357^* mutant line, in which a point mutation prevents binding of the receptor to the hormone-responsive elements, reveal an intricate network of GC-dependent transcription. Particularly, we show that Stat3 transcriptional activity mainly relies on glucocorticoid receptor GR tethering activity: several Stat3 target genes are induced upon glucocorticoid GC exposure both in wild type and in *gr^s357/s357^* larvae, but not in *gr* knock-out zebrafish. To understand the interplay between GC, their receptor, and the mineralocorticoid receptor, which is evolutionarily and structurally related to the GR, we generated an *mr* knock-out line and observed that several GC-target genes also need a functional mineralocorticoid receptor MR to be correctly transcribed. All in all, zebrafish mutants and transgenic models allow in vivo analysis of GR transcriptional activities and interactions with other transcription factors such as MR and Stat3 in an in-depth and rapid way.

## 1. Introduction

The glucocorticoid receptor (GR) is encoded by the *NR3C1* gene and normally localized in the cytoplasm in a multimeric complex composed of heat shock protein (HSP) 70 and 90 and immunophilins. When glucocorticoids (GCs) bind to GR, the FK506-binding protein 51 (FKBP51) is substituted by FKBP52 and dynein is recruited to allow the migration of GC/GR in the nucleus [1]. Once in the nucleus, GR detaches from HSP90 [2] and regulates the transcription of target genes directly interacting with DNA at the level of hormone response elements (HRE). HREs are cis elements shared with other steroid receptors such as the mineralocorticoid receptor (MR), the androgen receptor (AR) or the progesterone receptor (PR) [3]. Additionally, GR can affect the activity of other transcription factors through tethering or complex protein–protein–DNA interactions described in Ratman et al. [4]. The lack of GR in mice is lethal: *Nr3c1* knock-out (KO) animals die after birth for severe delay of lung development [5]. On the other hand, *Gr^dim/dim^* mice, characterized by the A458T mutation in the second zinc finger that abrogates GR dimerization and the subsequent HRE-dependent transactivation, are vital and reach adulthood, hence underlining alternative GR-dependent mechanisms of transcription activation [6]. Although GR monomers expressed by *Gr^dim/dim^* mice mutants have been proven to bind DNA efficiently [7,8], recent data obtained by Johnson and collaborators demonstrated that GR monomers poorly bind to chromatine and induce the transcription of a restricted number of GC-related genes [9].

However, with reduced transcriptional activity, GR monomers can indeed bind several transcription factors such as nuclear factor κ-light-chain-enhancer of activated B cells (NF-κB), signal transducer and activator of transcription 3 (STAT3) and activator protein-1 (AP-1) [10,11] and thus the *Gr^dim/dim^* model can allow discriminating between GR activity as dimer or monomer.

As recently reviewed by Dinarello et al. [12], in the last 20 years, zebrafish has gained importance for the study of GC/GR activities and several mutants have been generated for the genes encoding GR or the enzymes involved in the synthesis of GCs. In zebrafish, GR is encoded by a single gene called *nr3c1*, which shares a high similarity with the human orthologue [13].

In this work, taking advantage of two zebrafish mutant lines, the *nr3c1^ia30^* [14] and the *nr3c1^s357^* [15,16], we decided to better investigate in vivo the different mechanisms used by GR in the regulation of gene transcription. The *nr3c1^ia30^* (herein called *gr^ia30/ia30^*) line has been generated with CRISPR/Cas9 technology and is characterized by an insertion of 5 nucleotides in the second exon of the *nr3c1* gene. This mutation leads to a frameshift and a premature stop codon, and the mutation affects the response to stress stimuli, the feeding entrainment of zebrafish circadian clock and reproduction [14,17,18]. On the other hand, the *nr3c1^s357^* mutant line, generated with N-ethyl-N-nitrosourea (ENU) mutagenesis, has a point mutation that prevents GR interaction with the DNA [19]. However, *nr3c1^s357/s357^* mutants (herein called *gr^s357/s357^*) can still synthesize an entire GR protein (replacing an arginine 443 with a cysteine in the second zinc finger) available for interaction with other possible proteins and, hence, retaining all its HRE independent transcriptional activities. Starting from the experimental evidence shown by Facchinello et al. [14] and Vettori et al. [20], we wanted to analyse in more detail the differences between these two *gr* mutant lines, aiming to show how their use can help to uncover the functions that GR accomplishes by directly interacting with the DNA and the processes affected through the regulation of other transcription factors.

A complete evaluation of GR activity as a transcription factor cannot disregard the role of MR, that, interacting with GR in heterodimeric or heterotetrametic complexes, seems to regulate GR-dependent activities [21,22], affecting the response to GCs at the transcriptional level. In detail, GR and MR can interact with each other [23,24] and the heterodimer can affect gene transcription, thus increasing the potential roles of GC in tissues such as the brain, that expresses both receptors [22,24,25]. Consequently, the MR:GR ratio is critical for the maintenance of neuronal functions [26] and its imbalance was connected to behavioural dysfunctions, cognitive disorders [27] and depression in humans [28,29,30]. The higher expression of MR in the teleost brain also suggests that, in zebrafish, MR has a major role in regulating stress axis behaviour [31,32].

The GR and MR collaboration in the regulation of target genes transcription is particularly interesting in teleosts. GR and MR are structurally, evolutionarily, and functionally tightly connected [33] but, while in mammals MR also binds a specific ligand, aldosterone, and regulates ion balance and homeostasis [21,34], it has been demonstrated that in zebrafish GR accomplishes these functions [35,36,37,38,39,40]. Hence, in zebrafish, MR exerts only cortisol-related activities and *Danio rerio* can be an excellent model to study all the MR/cortisol-dependent processes that are also conserved in mammals, such as obesity, heart failure and depression [21]. For these reasons and to better elucidate the interplay between GR and MR, we decided to generate a new *mr* (*nr3c2*) KO zebrafish line with CRISPR/Cas9 technology, to investigate the role of MR in the regulation of GC/GR-dependent transcription.

## 2. Results

### 2.1. The Expression of GC-Dependent Genes klf9, epas1a and ucp2 Confirms Differences between gr^ia30/ia30^ and gr^s357/s357^ Mutants’ GCs Response

**Figure 1:**
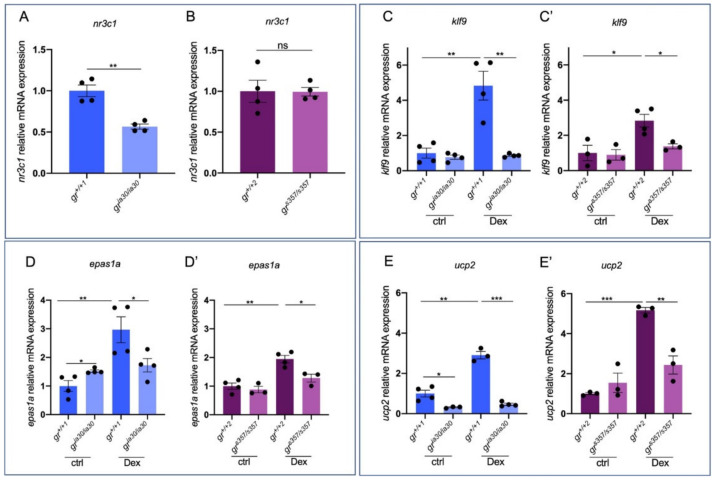
GR direct target genes show slight differences of expression between *gr^ia30^* and *gr^s357^* mutant lines. (**A**) RT-qPCR analysis of *nr3c1* in 6 dpf *gr^+/+1^* and *gr^ia30/ia30^* larvae. (**B**) RT-qPCR analysis of *nr3c1* in 6 dpf *gr^+/+2^* and *gr^s357/s357^* larvae. (**C**) RT-qPCR analysis of *klf9* in 6 dpf *gr^+/+1^* and *gr^ia30/ia30^* (**C**) and in *gr^+/+2^* and *gr^s357/s357^* (**C’**) larvae with or without Dex treatment. (**D**) RT-qPCR analysis of *epas1a* in 6 dpf *gr^+/+1^* and *gr^ia30/ia30^* (**D**) and in *gr^+/+2^* and *gr^s357/s357^* (**D’**) larvae with or without Dex treatment. (**E**) RT-qPCR analysis of *ucp2* in 6 dpf *gr^+/+1^* and *gr^ia30/ia30^* (**E**) and in *gr^+/+2^* and *gr^s357/s357^* (**E’**) larvae with or without Dex treatment. Statistical analyses were performed with Student’s *t* test. Mean ± SEM. * *p* < 0.05; ** *p* < 0.01; *** *p* < 0.001.

Prior to the analysis of the differences between the two *gr* mutant lines at the transcriptional level, we confirmed the downregulation of *nr3c1* mRNA expression only in *gr^ia30/ia30^* compared to *gr^+/+^* siblings (named *gr^+/+1^*, to distinguish them from *gr^+/+2^*, siblings of *gr^s357/s357^* larvae) (Figure 1A,B). Therefore, only in the *gr^ia30/ia30^* mutants the protein is absent or, when present, it is truncated and completely non-functional.

Mainly, our experimental approach was based on RT-qPCR analysis of the expression of GC-dependent genes in basal conditions or after 6 h of treatment with the synthetic GC Dexamethasone (Dex).

Since both lines, *gr^ia30/ia30^* and *gr^s357/s357^*, lack a GR that can bind to HREs (respectively due to *gr* KO and to the point mutation in GR DNA-binding domain), we found the expression of GR direct target genes significantly dampened in both lines. As already reported by Facchinello and collaborators [14], two important GC/GR target genes like *fk506-binding protein 5* (*fkbp5*), which belongs to the negative feedback loop induced by GC/GR for self-regulation, and *forkhead box protein O 3b* (*foxo3b*), which is upregulated by GCs [41], are significantly downregulated in both mutant lines compared to wild type (WT) and, in both *gr^ia30/ia30^* and *gr^s357/s357^* homozygous mutants, Dex treatment does not induce the expression of these two genes.

Here, as reported in Figure 1C,D, we confirmed the previous results by analysing the expression of other two GC/GR-dependent genes: *krueppel-like factor 9* (*klf9*), whose GC-dependent induction has a pro-inflammatory effect upon prolonged cortisol exposure [42], and *endothelial PAS domain protein 1a* (*epas1a*), a GC-regulated gene that encodes for hypoxia-inducible factor 2α (HIF2α) [43,44]. These results demonstrate that both mutant lines cannot induce the expression of *klf9* and *epas1a* when exposed to exogenous GCs, confirming that HRE-related gene transcription cannot be properly induced in both mutant lines. Of note, in *gr^ia30/ia30^* line, at the basal level the *epas1a* expression is significantly upregulated, suggesting that the high levels of cortisol in *gr^ia30/ia30^* larvae [14] can determine the upregulation of GC-target genes probably acting via other steroid hormone receptors, such as the MR that has a high affinity for cortisol [26].

Furthermore, we measured the level of expression of *uncoupling protein 2* (*ucp2*) that encodes for a proton transporter [45] and allows the GCs regulation of mitochondrial biogenesis in muscle [46]. Interestingly, it is severely downregulated in *gr^ia30/ia30^* compared to *gr^+/+1^*. On the other hand, no significant differences were observed between *gr^+/+2^* and *gr^s357/s357^*, thus revealing a basal differential expression of *ucp2* in *gr^ia30/ia30^* and *gr^s357/s357^*. While a 6 h long treatment with Dex in *gr^+/+1^* determines an upregulation of this gene, both mutant lines appeared to be insensitive to Dex treatment since we could not detect significant differences in *ucp2* expression levels between treated and untreated mutants, suggesting that this gene is properly activated via the DNA-binding domain (Figure 1E).

These results confirmed that direct DNA-binding-dependent GC-induced transcription is significantly dampened in these two lines, even if some small differences are evident.

### 2.2. Analysis of GC-Dependent Transcription in mr Mutant Zebrafish Larvae 

As mentioned above, MR is an important modulator of GR activity for its capability to regulate GC-dependent transcription generating MR/GR heterodimers and heterotetramers and to bind HRE promoter regions on DNA. MR is encoded by the *nr3c2* gene and in zebrafish has a high affinity for cortisol and for 11-deoxycorticosterone (DOC) [31]. To test whether MR has a function in the different expression patterns of *gr^ia30^* and *gr^s357^* zebrafish lines and to evaluate the possible roles of MR/GR interaction in the expression of GC-target genes, we decided to generate a new *nr3c2* zebrafish mutant line with CRISPR/Cas9 approach. The *nr3c2^ia32/ia32^* (herein called *mr^ia32/ia32^*) zebrafish mutant line is characterized by a deletion of 11 nucleotides in the third exon of the *nr3c2* gene. This mutation determines a frameshift with the subsequent generation of a premature stop codon (Figure S1A–D). Homozygous mutants are predicted to encode an aberrant, truncated protein of 628 amino acids, 17 of which are determined by the mutation. The mutated MR lacks the DBD, the LBD and the activation domain in the C-terminus (Figure S1A).

**Figure 2:**
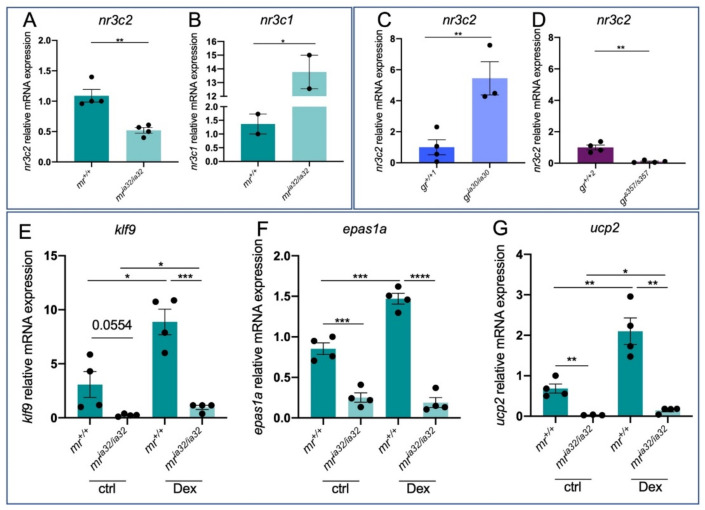
Analysis of GC-dependent genes in *mr* mutant zebrafish larvae. (**A**) RT-qPCR analysis of *nr3c2* in 6 dpf *mr^+/+^* and *mr^ia32/ia32^*. (**B**) RT-qPCR analysis of *nr3c1* in 6 dpf *mr^+/+^* and *mr^ia32/ia32^*. (**C**) RT-qPCR analysis of *nr3c2* in 6 dpf *gr^+/+^* and *gr^ia30/ia30^*. (**D**) RT-qPCR analysis of *nr3c2* in 6 dpf *gr^+/+^* and *gr^s357/s357^*. (**E**) RT-qPCR analysis of *klf9* in 6 dpf *mr^+/+^* and *mr^ia32/iaq32^* larvae with or without Dex treatment. (**F**) RT-qPCR analysis of *epas1a* in 6 dpf *mr^+/+^* and *mr^ia32/ia32^* larvae with or without Dex treatment. (**G**) RT-qPCR analysis of *ucp2* in 6 dpf *mr^+/+^* and *mr^ia32/ia32^* larvae with or without Dex treatment. Statistical analyses were performed with Student’s *t* test. Mean ± SEM. * *p* < 0.05; ** *p* < 0.01; *** *p* < 0.001; **** *p* < 0.0001.

Moreover, homozygous mutants are also characterized by a significant reduction of *nr3c2* mRNA expression compared with *mr^+/+^* siblings (Figure 2A), possibly due to the nonsense-mediated mRNA decay (NMD) commonly activated in KO zebrafish lines [47]. *mr^+/+^*, *mr^+/ia32^* and *mr^ia32/ia32^* animals can be easily distinguished by PCR with the primers listed in Table 1 (Figure S1E). Homozygous mutants are viable and reach adulthood, even if the mutation has some effects on survival: as reported in Figure S1F, the percentage of mutants observed at one-month post-fertilization is significantly lower (17%) than the expected one (25%). Interestingly, *nr3c1* mRNA expression is more upregulated in *mr^ia32/ia32^* larvae than *mr^+/+^* siblings (Figure 2B), suggesting that compensation mechanisms are triggered in this mutant line. Of note, *nr3c2* mRNA levels are significantly higher in *gr* homozygous mutants compared to WT siblings, while this transcript is significantly downregulated in *gr^s357/s357^* mutants compared to WT (Figure 2C,D), showing another substantial difference between these two *gr* mutant lines.

The upregulation of *nr3c1* transcript and the possible compensatory effect that characterizes *mr^ia32/ia32^* mutants is further supported by the higher fluorescence intensity of *mr^ia32/ia32^* and *mr^+/ia32^* larvae in *Tg(9xGCRE-Hsv.Ul23:EGFP)^ia20^* background (the zebrafish reporter line of GC-related transcription described by Benato et al. [48]) when compared to *mr^+/+^* siblings (Figure S2A). This phenomenon probably derives from the increased expression of *nr3c1* mRNA in mutants compared to WT since, as reported by Faught and Vijayan [32] with their *mr^ca402^* zebrafish KO line, the lack of Mr does not lead to hypercortisolemia, a feature that is further confirmed in the *mr^ia32^* line, where the levels of expression of *pomca* are not affected in *mr* mutants compared to wild type siblings (Figure S2B). 

Then, we tested the expression levels of the genes previously analysed in *gr* mutants, comparing *mr^+/+^* and *mr^ia32/ia32^* larvae and testing their responsiveness to Dex. Interestingly, *mr* KO determines a strong insensitivity to exogenous GCs since the expression levels of *klf9*, *epas1a* and *ucp2* are significantly different in treated *mr^ia32/ia32^* compared to treated *mr^+/+^* (Figure 2E–G). Additionally, it is worth noting that the lack of a functional MR significantly affects the basal expression of *epas1a* and *ucp2*. These results suggest that MR is essential for the correct GC-dependent transcriptional response and that it exerts many functions in the regulation of hypoxia response and mitochondrial homeostasis.

**Figure 3:**
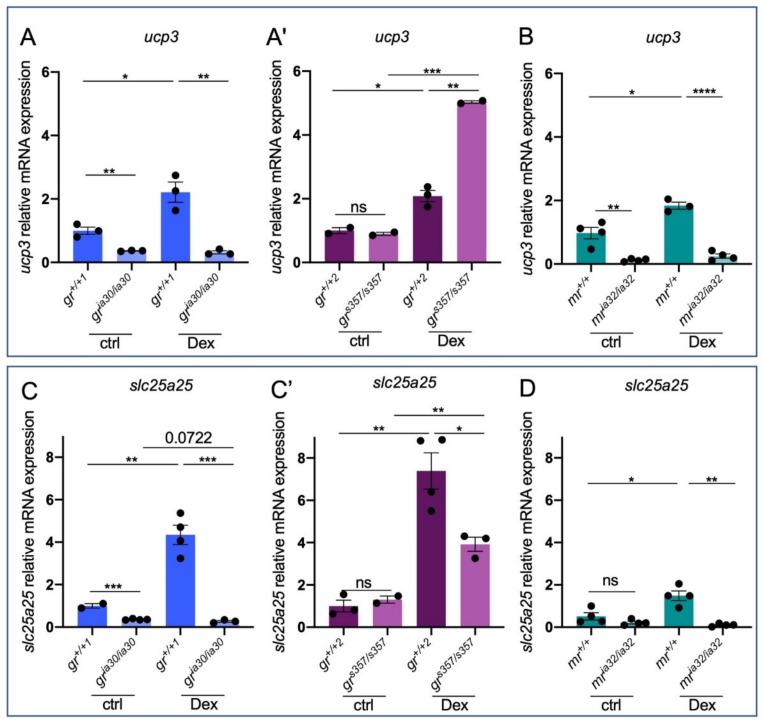
GC-dependent genes reveal different expression levels in the two *nr3c1* zebrafish mutant lines. (**A**) RT-qPCR analysis of *ucp3* in 6 dpf *gr^+/+1^* and *gr^ia30/ia30^* (**A**) and in *gr^+/+2^* and *gr^s357/s357^* (**A’**) larvae with or without Dex treatment. (**B**) RT-qPCR analysis of *ucp3* in 6 dpf *mr^+/+^* and *mr^ia32/ia32^* larvae with or without Dex treatment. (**C**) RT-qPCR analysis of *slc25a25* in 6 dpf *gr^+/+1^* and *gr^ia30/ia30^* (**C**) and in *gr^+/+2^* and *gr^s357/s357^* (**C’**) larvae with or without Dex treatment. (**D**) RT-qPCR analysis of *slc25a25* in 6 dpf *mr^+/+^* and *mr^ia32/ia32^* larvae with or without Dex treatment. Statistical analyses were performed with Student’s *t* test. Mean ± SEM. * *p* < 0.05; ** *p* < 0.01; *** *p* < 0.001; **** *p* < 0.0001; ns = not significant.

To better investigate this last aspect, we tested the expression of other mitochondrial proteins in the mutant lines of interest. We analysed the level of expression of *ucp3* and *solute carrier family 25 member 25* (*slc25a25*). As shown in Figure 3, these transcripts are upregulated by Dex in *gr^+/+^* and *mr^+/+^* larvae. However, while *gr^ia30/ia30^* show a basal lower level of expression of these transcripts compared to *gr^+/+1^* siblings and appear to be totally insensitive to exogenous GCs (Figure 3A,C), *gr^s357/s357^* larvae do not show significant differences compared to *gr^+/+2^* and Dex upregulates the expression of these genes either in *gr^+/+2^* and in *gr^s357/s357^* (Figure 3A’,C’). It is worth mentioning that all these transcripts are significantly downregulated in *gr^ia30/ia30^* compared to *gr^+/+1^* in basal conditions and we could not see any statistically significant difference between *gr^+/+2^* and *gr^s357/s357^*, suggesting that the two mutant lines have different transcriptional profiles in basal conditions. Considering also the expression profiles of the same genes in the *mr^ia32/ia32^* larvae, only *ucp3* showed a reduction in basal expression in *mr^ia32/ia32^* larvae if compared to *mr^+/+^* siblings (Figure 3B,D). Interestingly, these genes do not show a response to Dex stimulation in *mr^ia32/ia32^*, as observed in *gr^ia30/ia30^* (Figure 3B,D). These results suggest that possibly GR regulates the expression of these genes in a DNA-binding independent way and the expression of the identified mitochondrial proteins is also connected to MR function.

### 2.3. gr^ia30/ia30^ and gr^s357/s357^ Zebrafish Lines Reveal DNA-Binding Independent Mechanisms of Regulation of Stat3 

**Figure 4:**
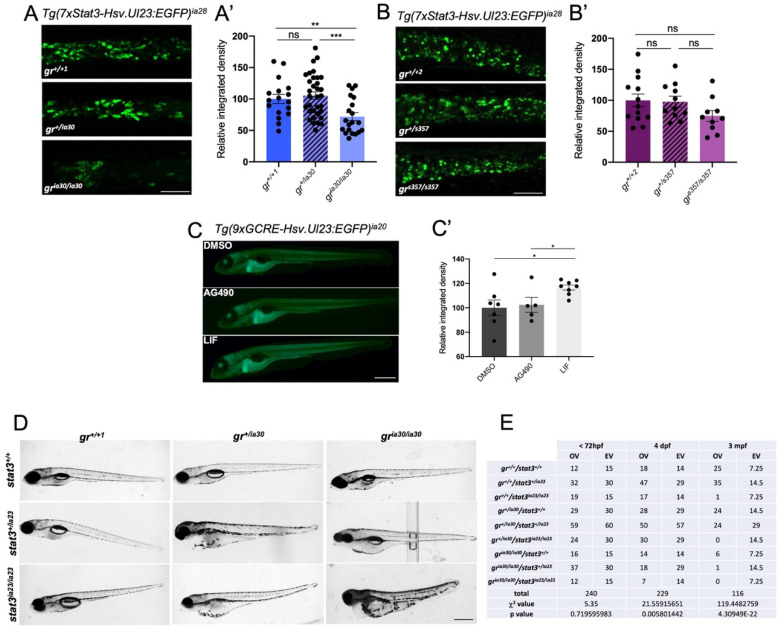
Analysis of crosstalk between GR and Stat3 with zebrafish mutant and transgenic lines. (**A**) Representative pictures (**A**) and fluorescence quantification (**A’**) of *Tg(7xStat3-Hsv.Ul23:EGFP)^ia28^* 6 dpf larvae at the level of the intestine in *gr^+/+1^*, *gr^+/ia30^* and *gr^ia30/ia30^* genetic background. Scale bar= 100 μm. (**B**) Representative pictures (**B**) and fluorescence quantification (**B’**) of *Tg(7xStat3-Hsv.Ul23:EGFP)^ia28^* 6 dpf larvae at the level of the intestine in *gr^+/+2^*, *gr^+/s357^* and *gr^s357/s357^* genetic background. Scale bar = 100 μm. (**C**) Representative pictures (**C**) and fluorescence quantification (**C’**) of *Tg(9xGCRE-Hsv.Ul23:EGFP)^ia20^* incubated from 3 to 6 dpf with DMSO, 50 μM AG490 and 20 μM LIF. Scale bar = 500 μm. (**D**) representative pictures of 6 dpf larvae generated by the breeding between *gr^+/ia30^/stat3^+/ia23^* zebrafish. Scale bar = 500 μm. (**E**) Table of observed (OV) and expected (EV) values of animals belonging to the 9 different genotypes obtained from breedings between *gr^+/ia30^/stat3^+/ia23^* zebrafish: χ^2^ test shows not significant differences between OV and EV in genotype distribution until 72 hpf (*p*-value = 0.7196); significant differences between OV and EV were detected at 4 dpf (** *p*-value = 0.0058) and 3 mpf (**** *p*-value = 4.30949 × 10^−22^). Mean ± SEM. Statistical analyses were performed with Student’s *t* test (A,B,C) and χ^2^ test (**E**). * *p* < 0.05; ** *p* < 0.01; *** *p* < 0.001; ns = not significant.

The tight connection between GR and other transcription factors has already been studied [49]. Specifically, the crosstalk of GR with STAT3 was widely described by Langlais and collaborators [50]. Here, for the first time with in vivo models, we decided to better elucidate how the GR–Stat3 crosstalk works and identify some Stat3-dependent transcripts which need GR to be properly activated. The analysis of the crosstalk between GR and Stat3 allowed us to highlight again the differences between *gr^ia30/ia30^* and *gr*^s357/s357^ zebrafish mutant lines. Firstly, taking advantage of the *Tg(7xStat3-Hsv.Ul23:EGFP)^ia28^* zebrafish reporter line that expresses EGFP in Stat3-positive cells [51], we tested whether Stat3-dependent transcription in intestinal stem cells is altered in *gr^ia30/ia30^* and *gr^s357/s357^* mutant lines. Interestingly, as reported in Figure 4A, *gr^ia30/ia30^;Tg(7xStat3-Hsv.Ul23:EGFP)^ia28^* 6 dpf larvae are characterized by a significantly lower intestinal fluorescence compared to *gr^+/+1^;Tg(7xStat3-Hsv.Ul23:EGFP)^ia28^* siblings. On the other hand, we could detect a slight but not significant difference in EGFP fluorescence between *gr^s357/s357^;Tg(7xStat3-Hsv.Ul23:EGFP)^ia28^* compared to *gr^+/+2^;Tg(7xStat3-Hsv.Ul23:EGFP)^ia28^* larvae (Figure 4B). This result suggests that Stat3 transcriptional activation is different in the two *gr* zebrafish mutant lines analysed and that GR-dependent regulation of Stat3 activity relies mainly on DNA-binding independent mechanisms of GR. Interestingly, 24 h long treatment with 10 μM Dex did not affect the fluorescence of Stat3 reporter in the intestine of WT larvae (Figure S3A). Furthermore, we also decided to see whether Stat3 signalling affects GR transcriptional activity. To do so, we analysed the levels of fluorescence of the GC zebrafish reporter line *Tg(9xGCRE-Hsv.Ul23:EGFP)^ia20^* [48] after chemical inhibition of the Stat3 pathway with AG490 (Figure 4C). No significant differences in reporter fluorescence were detected, meaning that the inhibition of Stat3 does not affect GC/GR-dependent transcription. To confirm this result, we also measured the fluorescence of *Tg(9xGCRE-Hsv.Ul23:EGFP)^ia20^* in *stat3* mutant background, taking advantage of a *stat3^ia23^* zebrafish mutant line [51,52]. No significant differences between *stat3^+/+^*, *stat3^+/ia23^* and *stat3^ia23/ia23^* 6 dpf larvae were observed (Figure S3B). Additionally, Dex treatment determines a significant increase of fluorescence in all the three genotypes analysed compared to untreated controls (Figure S3B). These results demonstrated that the lack of Stat3 does not significantly interfere with the nuclear activities of the GC/GR pathway. Moreover, a further confirmation of this result came from RT-qPCR analysis of *fkbp5* performed on *stat3^+/+^*, *stat3^+/ia23^* and *stat3^ia23/ia23^* after Dex treatment: we could not detect significant differences in *fkbp5* expression between *stat3^+/+^, stat3^+/ia23^* and *stat3^ia23/ia23^* and Dex treatment significantly induced the expression of this gene in all the genotypes under investigation (Figure S3C). However, it is worth noting that 3-day long treatment of *Tg(9xGCRE-Hsv.Ul23:EGFP)^ia20^* with leukaemia inhibitory factor (LIF), an activator of the Jak/Stat3 pathway that upregulates the intestinal fluorescence of Stat3 reporter line (Figure S3D), determines a significant upregulation of GC reporter fluorescence (Figure 4C,C’), demonstrating that the overstimulation of the Jak/Stat3 pathway determines the activation of GR transcriptional activity.

Further data showing the different impacts of *gr^ia30^* and *gr^s357^* mutations on Stat3 are shown in Figure 4D. We bred *gr^+/ia30^* mutants with *stat3^+/ia23^* larvae and subsequently, we performed *gr^+/ia30^/stat3^+/ia23^* double heterozygotes crosses to obtain the 9 possible genotypes. Interestingly, *gr^ia30/ia30^/stat3^ia23/ia23^* double homozygous mutants are characterized by severe developmental defects like large cardiac oedema, lack of yolk absorption, small head size and start dying at 4 dpf (Figure 4D,E). The severe phenotype of these double mutants was expected, since the roles of Stat3 and GR in development are well-known [53,54]. Interestingly, the defects described above were also detected in *gr^ia30/ia30^/stat3^+/ia23^*, *gr^+/ia30^/stat3^ia23/ia23^* and in some of *gr^+/ia30^/stat3^+/ia23^* larvae. Notably, *gr^ia30/ia30^/stat3^+/ia23^* and *gr^+/ia30^/stat3^ia23/ia23^* cannot reach adulthood (Figure 4D,E). Finally, in five breedings between double heterozygous, only three adult *gr^ia30/ia30^/stat3^+/ia23^* have been observed reaching the humane endpoint at 3 months of age. Conversely, the offspring generated by the breeding between *gr^+/s357^/stat3^+/ia23^* double heterozygotes adult zebrafish did not show any morphological defects and adult *gr^s357/s357^/stat3^+/ia23^* have been detected with the expected frequency (12.5%). These results confirmed that GR and Stat3 activities are tightly connected and that GR DNA-binding properties are not required for Stat3 activation and the proper induction of Stat3 transcription.

### 2.4. GR Regulates Stat3-Transcriptional Activities in DNA-Binding Independent Mechanisms with a Contribution of MR

Further investigations of GR-dependent control of Stat3 transcriptional activity were performed with RT-qPCR analysis of Stat3 target genes in both *gr^ia30/ia30^* and *gr^s357/s357^* 6 dpf zebrafish larvae. We analysed the levels of expression of *suppressor of cytokine signalling-3a* (*socs3a*), *hypoxia-inducible factor 1α like* (*hif1αl*), *unc-51 like autophagy activating kinase 2* (*ulk2*), *patatin-like phospholipase 3* (*pnpla3*) and *DNA damage-inducible transcript 4* (*ddit4*). *socs3a* is a Stat3 target gene that represses the Jak/Stat3 pathway and binds Janus Kinases (Jaks), inhibiting their function as an activator of Stat3 [55]. The *hif1αl* gene encodes for hypoxia-inducible factor 3α (Hif3α), a factor involved in hypoxia response—a process in which both GR and Stat3 are well-established players [20,56,57,58,59,60]. In addition, its promoter is predicted to harbour Stat3 binding elements (https://maayanlab.cloud/Harmonizome/ accessed on 12 September 2021). Ulk2 is an autophagy inducing kinase and its transcription is positively modulated by Stat3 [61]. *pnpla3* encodes for an enzyme that has hydrolase activity on retinyl esters and triglycerides [62] whereas *ddit4* is upregulated in response to different stressors and its transcription is induced by GCs [63] and phosphorylated Stat3 [64]. 

**Figure 5:**
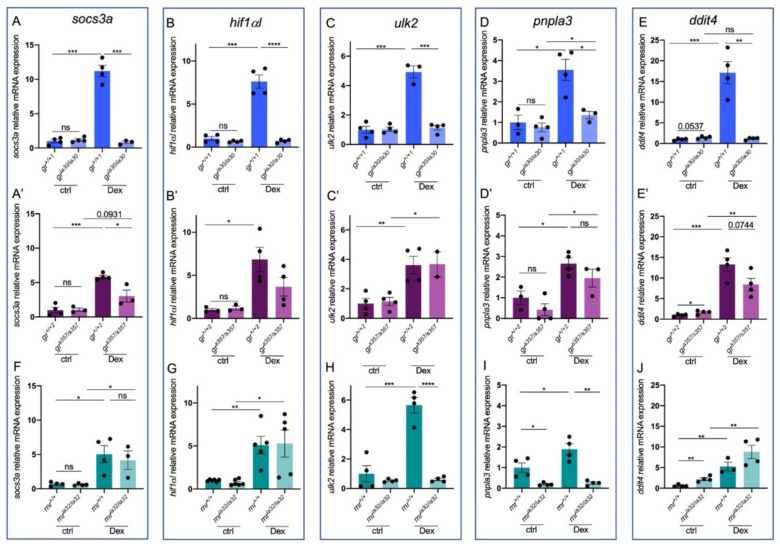
Stat3-dependent genes are differentially expressed in *gr^ia30^* and *gr^s357^* mutant lines. (**A**) RT-qPCR analysis of *socs3a* in 6 dpf *gr^+/+^* and *gr^ia30/ia30^* (**A**) and in *gr^+/+^* and *gr^s357/s357^* (**A’**) larvae with or without Dex treatment. (**B**) RT-qPCR analysis of *hif1αl* in 6 dpf *gr^+/+^* and *gr^ia30/ia30^* (**B**) and in *gr^+/+^* and *gr^s357/s357^* (**B’**) larvae with or without Dex treatment. (**C**) RT-qPCR analysis of *ulk2* in 6 dpf *gr^+/+^* and *gr^ia30/ia30^* (**C**) and in *gr^+/+^* and *gr^s357/s357^* (**C’**) larvae with or without Dex treatment. (**D**) RT-qPCR analysis of *pnpla3* in 6 dpf *gr^+/+^* and *gr^ia30/ia30^* (**D**) and in *gr^+/+^* and *gr^s357/s357^* (**D’**) larvae with or without Dex treatment. (**E**) RT-qPCR analysis of *ddit4* in 6 dpf *gr^+/+^* and *gr^ia30/ia30^* (**E**) and in *gr^+/+^* and *gr^s357/s357^* (**E’**) larvae with or without Dex treatment. (**F**) RT-qPCR analysis of *socs3a* in 6 dpf *mr^+/+^* and *mr^ia32/ia32^* larvae with or without Dex treatment. (**G**) RT-qPCR analysis of *hif1αl* in 6 dpf *mr^+/+^* and *mr^ia32/ia32^* larvae with or without Dex treatment. (**H**) RT-qPCR analysis of *ddit4* in 6 dpf *mr^+/+^* and *mr^ia32/ia32^* larvae with or without Dex treatment. (**I**) RT-qPCR analysis of *ulk2* in 6 dpf *mr^+/+^* and *mr^ia32/ia32^* larvae with or without Dex treatment. (**J**) RT-qPCR analysis of *pnpla3* in 6 dpf *mr^+/+^* and *mr^ia32/ia32^* larvae with or without Dex treatment. Statistical analyses were performed with Student’s *t* test. Mean ± SEM. * *p* < 0.05; ** *p*< 0.01; *** *p* < 0.001; **** *p* < 0.0001; ns = not significant.

*socs3a* and *hif1αl* expression are not different between *gr^ia30/ia30^* and *gr^s357/s357^* mutants and their respective WT siblings, meaning that, in basal conditions, they are not differentially expressed in the two genetic backgrounds. Additionally, they are significantly induced in *gr^+/+^* upon Dex treatment, confirming their GC-based transcriptional regulation. However, while *gr^ia30/ia30^* appeared to be completely insensitive to exogenous GCs (Figure 5A,B), we could observe a not significant increase in *socs3a* and *hif1αl* expression in *gr^s357/s357^* larvae upon Dex treatment (Figure 5A’,B’). Evidence about the differential control of Stat3 target gene expression in the two *gr* mutant lines came from the analysis of *ulk2*, *pnpla3* and *ddit4*. Dex significantly induces the expression of these three genes in *gr^+/+1^*, *gr^+/+2^* and *gr^s357/s357^*, when compared to untreated siblings, but had no effect on *gr^ia30/ia30^* larvae (Figure 5A–E,C’–E’). These results demonstrate that GCs regulate the expression of some Stat3-related genes mainly in a GR–DNA binding independent mechanism.

Moreover, we sought to assess whether the differential control of Stat3 target genes observed in *gr^ia30/ia30^* and in *gr^s357/s357^* can be due to compensating effects by MR. A first analysis of the *mr* mutant in *Tg(7xStat3-Hsv.Ul23:EGFP)^ia28^* Stat3 reporter line background demonstrated that, at least in the intestine, the lack of a functional MR does not affect Stat3-dependent transcription (Figure S2C). However, RT-qPCR analysis of the genes already reported in Figure 4 demonstrated that the absence of a functional MR partially affects the GC-mediated control of Stat3-related genes. As reported in Figure 5, the responsiveness of *mr^ia32/ia32^* for Dex is not significantly affected as regards the expression levels of *socs3a, hif1αl* and *ddit4*, since we could not see significant differences in their expression in treated *mr^ia32/ia32^* compared to treated *mr^+/+^*. However, *ulk2* and *pnpla3* mRNA expression is not upregulated in mutants, revealing that *mr^ia32/ia32^* show defects in the correct expression of Stat3-target genes and demonstrating that MR plays a role in this process together with GR. These transcriptional patterns show that some of these Stat3-dependent genes are controlled by GR alone (like *socs3a*, *hif1αl* and *ddit4*), but other genes, like *pnpla3* and *ulk2*, need both GR and MR to be properly induced. Of note, the basal upregulation of *ddit4* in *mr^ia32/ia32^* compared to *mr^+/+^* can be due to the significant upregulation of *nr3c1* that characterizes *mr* KO animals (Figure 2B).

## 3. Discussion

In this work, we decided to investigate the possibility of in vivo distinction between the different GCs-GR mechanisms of transcription regulation. Although the *gr^s357/s357^* zebrafish mutant line has been used as a KO model by several research groups [16,20,65,66,67,68,69,70], our previous works have already suggested that DNA-binding independent mechanisms are still present in the *gr^s357/s357^* [14,20]. GR transcriptional activity mechanisms have been debated for a long time. The protein works as a transcription factor in a homodimeric complex binding DNA in several different conformations [71], but some research has also shown that GR monomers can interact with DNA activating the transcription of a small set of target genes [7,8,9]. Notably, GR activity relies on several phosphorylations that regulate its interactions with DNA or with other proteins [72] and, recently, GR tetramers have been identified [73,74].

To gain new insights on tethering activity or complex protein–protein–DNA interactions of GR, Escoter-Torres and collaborators [75] generated a mutant mouse line carrying a point mutation in the first zinc finger of the DBD (converting cysteine 437 to glycine). This GR protein cannot bind to DNA, but still maintains the possibility of regulating transcription via protein–protein interactions. Of note, similarly to the GR null mice [5], this model (called *GR^∆Zn^*) is not viable since the embryos die for respiratory failure [75]. Consequently, all studies with both mouse mutant lines need to be performed in vitro, losing the integration of the in vivo models.

Here, with our zebrafish genetic models, we identified functional interactions of zebrafish GR with the MR in the regulation of several GC- and Stat3-related genes and demonstrated that DNA-binding-dependent properties of GR are not essential for inducing the transcription of a portion of GC-dependent genes. Although some canonical GC-responsive genes (such as *klf9*, *epas1a* and *ucp2*) are regulated by GR direct interaction with HREs and do not respond to Dex treatment in both mutants (*gr^ia30/ia30^* and *gr^s357/s357^*), other key GC-related genes have different expression profiles in the two zebrafish lines.

The different features of these mutants helped us to in vivo reveal that GR regulates Stat3 transcriptional activity mainly in a DNA-binding independent way. The expression analysis of some Stat3 targets, *hif1αl*, *ulk2*, *pnpla3* and *ddit4*, showed both that these Stat3 targets are Dex-responsive, and that their responsiveness is independent from GR–DNA-binding: the Dex-related upregulation of these genes was detected in treated *gr^+/+^* and *gr^s357/s357^* but not in *gr^ia30/ia30^*, thus confirming a substantial difference between *gr^ia30/ia30^* and *gr^s357/s357^*. We suppose that the GR regulation of Stat3 activity is due to nuclear interactions between these two transcription factors, a tethering mechanism that can control the expression of different target genes without the interaction of GR with HREs [50]. This hypothesis was further confirmed by both the severe phenotype of *gr^ia30/ia30^/stat3^ia23/ia23^* larvae, not detected in *gr^s357/s357^/stat3^ia23/ia23^* mutants, as well as the inability of the *gr^ia30/ia30^/stat3^+/ia23^* and *gr^+/ia30^/stat3^ia23/ia23^* to reach adulthood.

Among the GC-responsive genes we analysed, *ucp2*, a member of the mitochondrial solute carrier 25 family largely involved in mitochondrial homeostasis, also controlled by Stat3 [76,77], is clearly downregulated in *gr^ia30/ia30^* compared to *gr^+/+1^* siblings. Of note, we could not observe significant differences in *ucp2* expression between *gr^+/+2^* and *gr^s357/s357^* (Figure 1) suggesting that its basal expression might be regulated by GR in a DNA-binding independent way. However, we did not observe a significant increase of *ucp2* expression after Dex treatment of *gr^s357/s357^*, implying that *ucp2* regulation by GC depends on the binding of GR to DNA. This trend represents an intermediate expression pattern between the other canonical GC-responsive genes (*klf9* and *epas1a*) and Stat3 canonical targets taken into consideration in our analysis (*hif1αl*, *ulk2*, *pnpla3*, *ddit4*), suggesting that the regulation of GC-related gene expression can be more complex and different between basal and stress conditions (here mimicked with Dex treatment). As observed by Petta et al., and Langlais et al. [49,50] (and recently reviewed by Timmermans and collaborators [78]), the control of transcription factors activities performed by GR can happen in several different conformations and GR participates in this process as a monomer, a dimer or even a tetramer. Notably, STAT3 can be regulated by tethering activities that are lost in *gr^ia30/ia30^* mutants but are partially conserved in *gr^s357/s357^*. Additionally, it has been reported that other steroid hormone receptors, among which is included MR, regulate and are regulated by STAT3 [79,80,81]. 

Moreover, we need to keep in mind that GCs can bind GR and MR: two different but related receptors that have a different affinity for corticosteroids. The higher affinity of MR allows the receptor to be occupied in basal conditions while the lower affinity of GR determines its activation in response to GCs-increase following the stress or in phase with the GCs-circadian increase [82].

Both receptors are members of the steroid receptor family and show a high level of similarity: they compete for the same ligands and share the same hormone-responsive elements (HREs) on DNA [21]. Hence, to better understand the mechanisms underlying the differential expression of the genes analysed in basal and GC-stimulated conditions in the *gr* mutant lines, we took into consideration MR and generated a new *mr^ia32/ia32^* zebrafish line. The homology between GR and MR and the similar mechanisms of action justify the compensation mechanism detected in both mutant lines: *gr^ia30/ia30^* and *mr^ia32/ia32^* showed an upregulated expression of respectively *nr3c2* and *nr3c1* transcripts when compared to WT.

Of note, GR and MR can form heterodimers with each other, regulating the expression of specific target genes probably with a different efficacy: more specifically, MR homodimers are the main complex working at low levels of GCs, while MR/GR heterodimers and GR homodimers formations are predominant in stress conditions [21]. This fact can be an interesting starting point for the interpretation of our data. In the *mr^ia32/ia32^* line, the expression of canonical GC-dependent genes *klf9* and *epas1a* is downregulated in basal conditions; on the contrary, in *gr^ia30/ia30^ klf9* expression does not show significant differences when compared to *gr^+/+1^* and *epas1a* seems to be even more expressed in *gr^ia30/ia30^* rather than *gr^+/+1^*. Interestingly, regarding these two genes, the transcriptional response to Dex is completely erased in both *gr* and *mr* KO lines. Hence, considering the model of Gomez-Sanchez and collaborators [16], we can hypothesize that in basal conditions the expression of *klf9* and *epas1a* is regulated by MR homodimers. For this reason, in *gr^ia30/ia30^* line the expression of *klf9* and *epas1a* is not impaired and the upregulation of *nr3c2* in *gr^ia30/ia30^* explains the higher levels of expression of *epas1a* in *gr* KO. Upon Dex treatments, the response is possibly driven by GR/MR heterodimers as the sensitivity of these two mutant lines to GCs is severely dampened. Additionally, *pnpla3* also showed expression profiles in mutant lines that are compatible with the mechanism of regulation described by Gomez-Sanchez et al. [21]: GR seems to be not involved in the regulation of their expression in basal conditions, while *mr^ia32/ia32^* line showed a downregulated expression of *pnpla3* in basal conditions suggesting that MR homodimers control their transcription. Furthermore, *ulk2* seems to be sensible to Dex stimulation in an MR and GR-dependent way. On the contrary, *hif1αl, ddit4* and *socs3a* do not seem to be regulated by MR: *socs3a* and *hif1αl* levels of expression in basal conditions are not impaired in *mr^ia32/ia32^* line, in both basal and stimulated conditions; in the same way, *ddit4* does not show a difference between *mr^+/+^* and *mr^ia32/ia32^* larvae. Moreover, the significant upregulation in *mr^ia32/ia32^* compared to WT suggests the dependency of *ddit4* to GR, since *mr* mutants are characterized by high levels of expression of *gr*. We can conclude that MR probably cooperates with GR in the regulation of some Stat3 targets, regulating both their basal expression (*pnpla3*) and Dex induced response (*pnpla3* and *ulk2*).

Starting from the interesting expression profile of *ucp2* in *gr^ia30/ia30^* and *gr^s357/s357^* zebrafish lines, we have investigated the expression pattern of other solute carrier family 25 members (*ucp3* and *slc25a25*) to clarify how both MR and GR could be involved in the regulation of mitochondrial homeostasis. These two targets are upregulated by exogenous GCs and their expression relies on GR: in basal conditions the expression of these transcripts is significantly downregulated in *gr* KO compared to WT, but not in *gr^s357/s357^* compared to *gr^+/+2^*, suggesting a basal control of their expression by DNA-binding independent mechanism. After Dex exposure, the scenario slightly changes, and expression profiles demonstrate a more intricate mechanism of regulation. While *ucp2* appears to be regulated by GR-DNA-binding dependent mechanisms, the response to Dex of *ucp3* and *slc25a25* in *gr^s357/s357^* is independent from GR-DNA-binding. Interestingly, *ucp2* and *ucp3* genes showed similar expression patterns in *gr^ia30/ia30^* and *mr^ia32/ia32^*, with a downregulation of transcripts in both basal and stimulated conditions compared to *gr^+/+1^* and *mr^+/+^* respectively, suggesting that both receptors can be involved in the regulation of mitochondrial functions. On the contrary, the basal expression of *slc25a25* in *mr^ia32/ia32^* is not impaired, while in *gr^ia30/ia30^* it is downregulated. Finally, transcriptional data suggest that both GR and MR are involved in Dex stimulation of *upc3* and *slc25a25* genes. The level of expression of the analysed genes is summarized in Table 2.

In conclusion, we demonstrated in vivo that GC-dependent genes are regulated through several mechanisms that rely on both GR and MR. As already known, GR exerts its transcriptional activities by both binding directly to HRE, but also by tethering with other transcription factors. Our models allowed us to verify in vivo the two different mechanisms and to demonstrate that Stat3 is one of the most important transcriptional partners of GR. Notably, GR regulates Stat3 transcriptional activity mostly in a DNA-binding independent way, even if we cannot exclude a marginal role of canonical GR transcriptional activities in the modulation of the Jak/Stat3 pathway. Finally, it is worth mentioning that mitochondrial homeostasis is heavily regulated by GR, confirming the strong involvement of this protein in mitochondria activities [83,84,85]. Notably, the GR effect on the mitochondrial genes here analysed seems to be GR–DNA-binding independent and needs MR to be properly determined. All in all, the results obtained highlight the central role of MR as a prominent regulator and enhancer of GR transcriptional activity and underline the potential of the *gr* KO and *gr^s357/s357^* mutants for deepening knowledge on GC-dependent transcriptional mechanisms.

## 4. Materials and Methods

### 4.1. Animal Husbandry and Zebrafish Lines

Animals were staged and fed as described by Kimmel et al. [86] and maintained in a large-scale aquaria system. Embryos were obtained after natural mating, raised in Petri dishes containing fish water (50×: 5 g NaHCO_3_, 39.25 g CaSO_4_, 25 g Instant Ocean for 1:l) and kept in a 12:12 light/dark cycle at 28 °C. All experimental procedures complied with European Legislation for the Protection of Animals used for Scientific Purposes (Directive 2010/63/EU).

*gr^ia30^* [14], *mr^ia32^* and *stat3^ia23^* [51] mutant lines are genotyped by PCR amplification and 3% agarose gel migration. *gr^s357^* animals are genotyped as described in Facchinello et al. [9]. The *Tg(7xStat3-Hsv.Ul23:EGFP)^ia28^* line and the *Tg(9xGCRE-Hsv.Ul23:EGFP)^ia20^* line have been respectively characterized by Peron et al. [51] and by Benato et al. [48]. 

### 4.2. Generation of mr Zebrafish Mutant Line

The generation of the *mr* mutant zebrafish line was performed using the CRISPR/Cas9-mediated genome editing. Briefly, the CHOPCHOP (https://chopchop.rc.fas.harvard.edu accessed on 26 January 2022) and E-CRISP (available at http://www.e-crisp.org/E-CRISP/ accessed on 26 January 2022) software were used to design the gene-specific guide RNA (sgRNA) (GAGGCGTCAGGATGCCACTACGG) to target *nr3c2* gene on exon 2 following the protocol described in Gagnon et al. [87]. The guide was subsequently produced using the protocol described in Gagnon et al. [87]. Fertilized eggs were injected with 1 nL of a solution containing 280 ng/μL of Cas9 (M0386T, New England Biolabs) and 3 pmol/μL of *nr3c2*-targeting sgRNA. Genomic DNA was extracted from 4 dpf injected larvae to test the presence of mutations and to confirm the activity of the Cas9 enzyme. Injected embryos were raised to adulthood and screened, by F1 genotyping, for germline transmission of the mutation. An F1 mutant carrier harbouring a deletion of 11 nucleotides was selected and bred with WT to obtain the F2 generation. The resulting heterozygous F2 individuals were incrossed, to obtain F3 generation that includes homozygous mutants.

### 4.3. Imaging

For in vivo imaging, transgenic larvae were anaesthetized with 0.04% tricaine, embedded in 1% low-melting agarose and mounted on a depression slide. Nikon C2 confocal system was used to acquire images from *Tg(7xStat3-Hsv.Ul23:EGFP)^ia28^* transgenic larvae. *Tg(9xGCRE-Hsv.Ul23:EGFP)^ia20^* transgenic larvae were mounted in 1% low-melting agarose and observed with a Leica M165 FC microscope equipped with a Nikon DS-Fi2 digital camera. All images were analysed with Fiji (ImageJ2, Madison, WI, USA) software and fluorescence integrated density was calculated setting a standard threshold on non-fluorescent samples as described in Facchinello et al. [88].

### 4.4. Animal Treatments

We exposed 6 dpf *gr^+/+^*, *gr^ia30/ia30^*, *gr^s357/s357^*, *mr^+/+^*, and *mr^ia32/ia32^* larvae to 10 μM Dex for 6 h and the treatments started at 3 p.m. At the end of the treatment, larvae were anaesthetized and sacrificed for RNA extraction. *Tg(9xGCRE-Hsv.Ul23:EGFP)^ia20^* reporter larvae were treated with 50 μM AG490, 10 μM Dex and 20 μM LIF from 3 to 6 dpf.

### 4.5. mRNA Isolation and Quantitative Real-Time Reverse Transcription PCR (RT-qPCR)

Total RNAs were extracted from pools of 20 larvae at 6 dpf with TRIzol reagent (Thermo Fisher Scientific, Waltham, MA, USA, 15596018) and incubated at 37 °C for 30 min with RQ1 RNase-Free DNase (Promega, M6101, Madison, WI, USA). cDNA synthesis was performed using random primers (Promega, C1181) and M-MLV Reverse Transcriptase RNase H (Solis BioDyne, 06-21-010000) according to the manufacturer’s protocol. qPCRs were performed in triplicate with SybrGreen method by means of CFX384 Touch-Real Time PCR Detection System and the 5x HOT FIREPol EvaGreen qPCR Mix Plus (Solis BioDyne, Tartu, Estonia, 08-36-00001) and *ube2a* and *actb* were used as internal standards in each sample. The amplification protocol consists of 95 °C for 14 min followed by 45 cycles at 95 °C for 20 s, 60 °C for 20 s and 72 °C for 25 s. Threshold cycles (Ct) and melting curves were generated automatically by CFX384 Touch-Real Time PCR Detection System (Biorad, Hercules, CA, USA) and results were obtained with the method described in Livak and Schmittgen [89]. Sequences of genes of interest primers are listed in Table 1.

### 4.6. Statistical Analysis 

Statistical analyses were performed using GraphPad Prism (GraphPad Software Inc., San Diego, CA, USA). Different experimental groups for RT-qPCR and fluorescence quantifications were compared using Student’s *t* test. Observed genotype distributions (OV) were compared to the expected values (EV) (+/+ = 25%; +/− = 50%; −/− = 25%) using χ^2^ test. * *p* < 0.05; ** *p* < 0.01; *** *p* < 0.001, **** *p* < 0.0001; ns = not significant.

## Figures and Tables

**Table 1:** List of primers (5′–3′ sequences) used for genotyping and for RT-qPCR.

Gene	Forward Sequence (5′–3′)	Reverse Sequence (5′–3′)	Use
*nr3c1*	ACCACTTCAAGCGGACAGAG	CCGGCTTCTGATCTTTCTGC	Genotyping
*nr3c2*	GACAGCCAAAGTGTGTCTGG	TGAGTCTTACCTTCTACCGCTC	Genotyping
*stat3*	GGCCTCTCTGATAGTGACCG	GCATTGTATAAAGCGCTACAGAG	Genotyping
*ube2a*	CATCATGGTCTGGAACGCTG	GAGGAAACGTCATATGTTGGAC	RT-qPCR
*nr3c1*	CAACACAATTACCTGTGTGCTG	CTTGACGTGCCTTTGACTTGC	RT-qPCR
*nr3c2*	CTGAGGCACACGTCTTCG	CAGCACAAAGGTAGTTGTGC	RT-qPCR
*klf9*	GACCGACTGCACGCATCC	TTTTGCACAGCCAGGCCAG	RT-qPCR
*epas1a*	CCTACGACATGGGCGAAATA	GTCGCCTCTTCAAACTCTGC	RT-qPCR
*ucp2*	CACTGGACACCGCAAAAGTT	CGTACCAAAGACCCCTCGAT	RT-qPCR
*slc25a25a*	CTGCCGAAAACATTCCCAA	CCTCCACCACATCCCAGTTA	RT-qPCR
*ucp3*	GTGATGAGGGGTGTTCGAGG	TAGGTTATCTGTCATGAGGTCG	RT-qPCR
*socs3a*	GGAAGACAAGAGCCGAGACT	GCGATACACACCAAACCCTG	RT-qPCR
*ulk2*	GAAAGCAGCTCAGCTTCTGG	TCTGTGAGGCGACGGCAC	RT-qPCR
*hif1* *al*	ATGGGTGAGGTATGGGTTCG	AGAGCACACTTACCCACACA	RT-qPCR
*pnpla3*	CCTCTGGACGACTCTGTGTT	CGGAAGGCAGGAGGGATTAA	RT-qPCR
*ddit4*	GACTCTGACTCCGACAACC	TTACACAACGCCTCTTCAGTG	RT-qPCR
*pomca*	TGTCGAGACCTCAGCACAG	TGCGAGGAGGTCGATTTGC	RT-qPCR
*fkbp5*	GTGTTCGTCCACTACACC	TCTCCTCACGATCCCACC	RT-qPCR

**Table 2:** Schematic overview of expression of genes analysed in different mutant backgrounds. Non-significant differences are represented with “=”, significant downregulation with “⇓” and significant upregulation with “⇑”.

Gene	*gr^ia30/ia30^* vs. *gr^+/+1^*		*gr^s357/s357^* vs. *gr^+/+2^*		*mr^ia32/ia32^* vs. *mr^+/+^*	
	ctrl	Dex	ctrl	Dex	ctrl	Dex
*klf9*	=	⇓	=	⇓	=	⇓
*epas1a*	⇑	⇓	=	⇓	⇓	⇓
*ucp2*	⇓	⇓	=	⇓	⇓	⇓
*ucp3*	⇓	⇓	=	⇑	⇓	⇓
*scl25a25a*	⇓	⇓	=	⇓	=	⇓
*socs3a*	=	⇓	=	⇓	=	=
*hif1αl*	=	⇓	=	=	=	=
*ulk2*	=	⇓	=	=	=	⇓
*pnpla3*	=	⇓	=	=	⇓	⇓
*ddit4*	=	⇓	⇑	=	⇑	⇑

## Data Availability

The data that support the findings of this study are available from the corresponding author upon reasonable request.

## Supplementary Materials

The following are available online at https://www.mdpi.com/article/10.3390/ijms23052678/s1.
