# Supplementary material for: Zebrafish Mutant Lines Reveal the Interplay between nr3c1 and nr3c2 in the GC-Dependent Regulation of Gene Transcription
Source: Int J Mol Sci. 2022 Feb 28;23(5):2678. doi: 10.3390/ijms23052678 (PMC8910431; doi:10.3390/ijms23052678)
Supplement: Supplementary file 1 [file ijms-23-02678-s001.zip › ijms-1594556-supplementary.pdf]

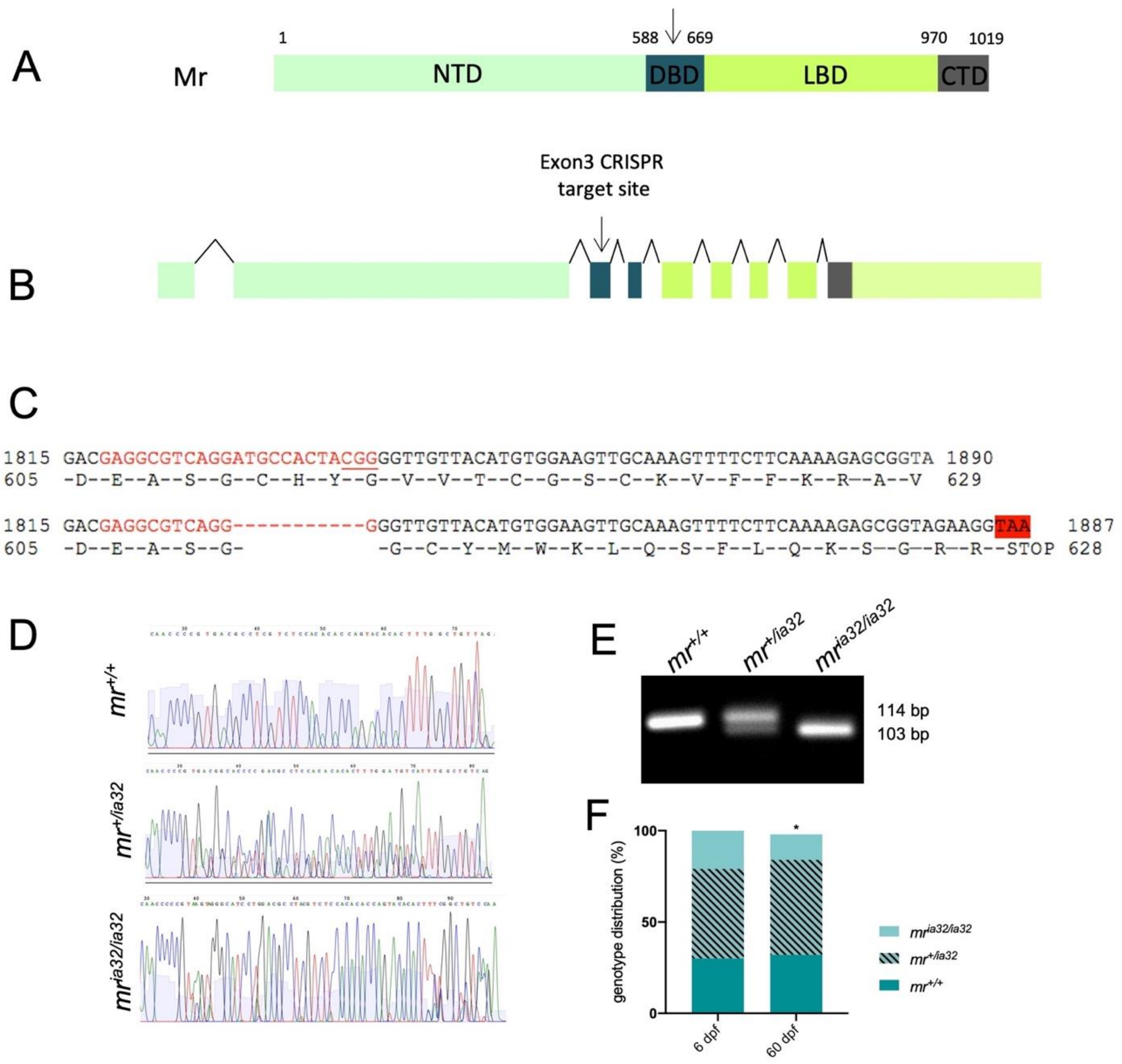

Figure S1. Generation of *mr* mutant line. (**A**) Schematic representation of zebrafish Mr protein composed of N-terminal domain (NTD), DBD, LBD and C-terminal domain (CTD). (**B**) Schematic representation of *nr3c2* exons. The arrow (both in A e B panel) indicates the region where the mutation occurred. (**C**) Partial alignment of WT and mutant *nr3c2* nucleotide and aminoacidic sequences. The premature stop codon (TAA) is highlighted in red. (**D**) chromatogram of $mr^{+/+}$, $mr^{+/ia32}$ and $mr^{ia32/ia32}$ DNA samples sequenced using the *nr3c2* reverse primer listed in Table 1. (**E**) Genotyping of *nr3c2* mutants with 3% agarose gel. (**F**) Survival rate of $mr^{+/+}$, $mr^{+/ia32}$ and $mr^{ia32/ia32}$ animals at 6 dpf (n=80 animals from 3 different breedings between $mr^{+/ia32}$ adults) and ad 60 dpf (n=80 animals from 3 different breedings between $mr^{+/ia32}$ adults). Statistical analyses were performed with χ^2 test. *p<0.05.

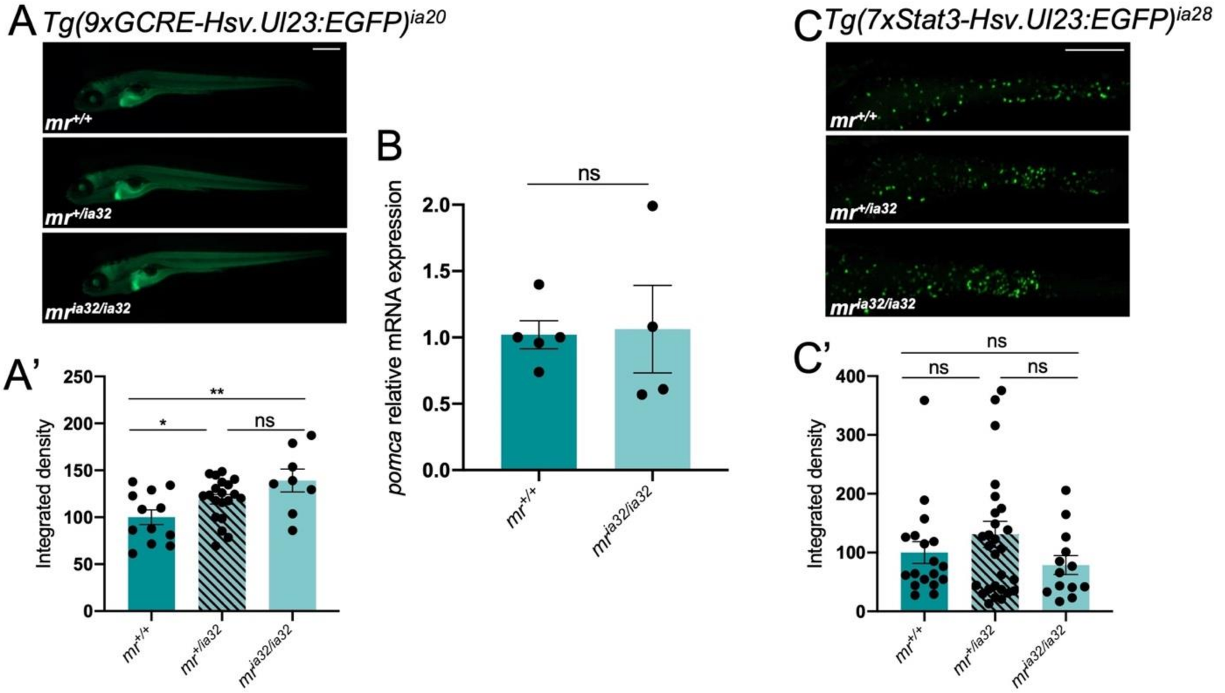

Figure S2. GC/Gr- and Stat3-dependent fluorescence in *mr* mutants. (**A**) Representative pictures (**A**) and fluorescence quantification (**A′**) of $mr^{+/+}$, $mr^{+/ia32}$ and $mr^{ia32/ia32}$ 6-dpf larvae in $Tg(9xGCRE\text{-}Hsv.Ul23:EGFP)^{ia20}$ transgenic background. Scalebar=500µm.(**B**) RT-qPCR of *pomca* mRNA in $mr^{+/+}$ and $mr^{ia32/ia32}$ 6-dpf larvae. (**C**) Representative pictures (**C**) and fluorescence quantification (**C′**) of $mr^{+/+}$, $mr^{+/ia32}$ and $mr^{ia32/ia32}$ 6-dpf larvae in $Tg(7xStat3\text{-}Hsv.Ul23:EGFP)^{ia28}$ transgenic backgroundintheintestine.Scalebar=100µm.Statisticalanalyseswereperformedwith Student *t* test. Mean ± SEM. *p<0.05; **p<0.001; ns = not significant.

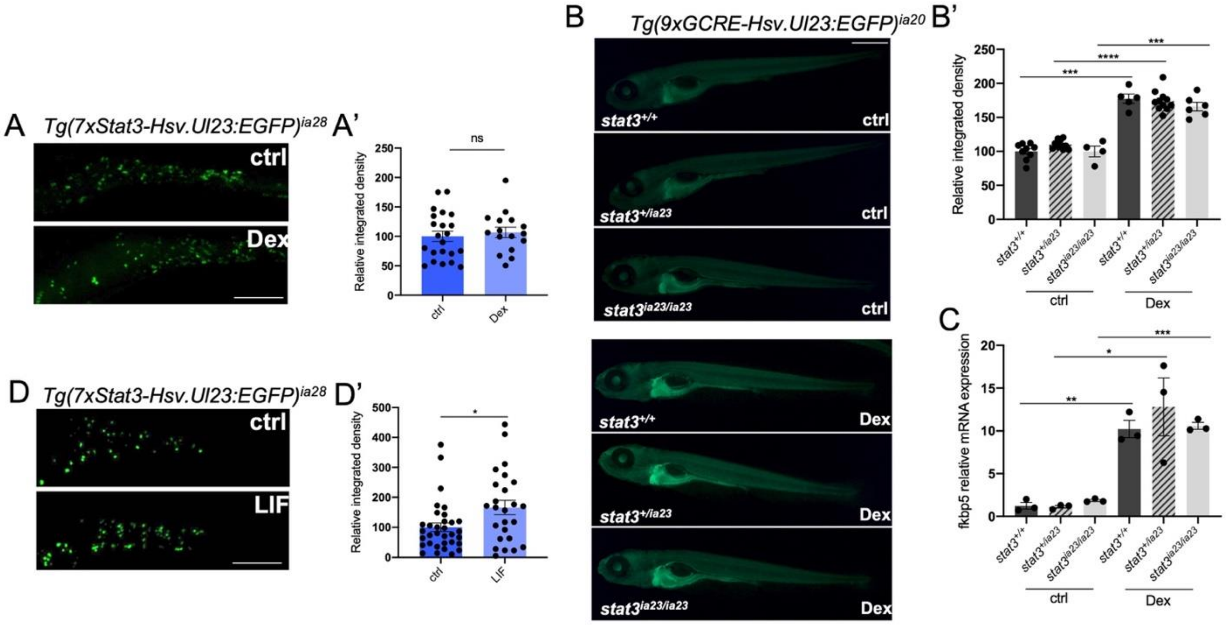

Figure S3. Gr-Stat3 crosstalk with transgenic lines. (**A**) Representative pictures (**A**) and fluorescence quantification (**A′**) of $Tg(7xStat3\text{-}Hsv.Ul23:EGFP)^{ia28}$ transgenic larvae intestine treated with Dex for 24hours.Scalebar=100µm.(**B**) Representative pictures (**B**) and fluorescence quantification (**B′**) of $stat3^{+/+}$, $stat3^{+/ia23}$ and $stat3^{ia23/ia23}$ in $Tg(9xGCRE\text{-}Hsv.Ul23:EGFP)^{ia20}$ transgenic background treated with Dex for24hours.Scalebar=500µm.(**C**) RT-qPCR analysis of

fkbp5 mRNA in 6-dpf s*tat3*$^{+/+}$, *stat3*$^{+/ia23}$ and *stat3*$^{ia23/ia23}$ treated with Dex for 24 hours. (**D**) Representative pictures (**D**) and fluorescence quantification (**D'**) of *Tg(7xStat3-Hsv.Ul23:EGFP)*^{ia28} transgenic larvae intestine treated with 20 μM LIF for 3 days. Scale bar = 100 μm. Statistical analyses were performed with Student *t* test. Mean ± SEM. *p<0.05; **p<0.01; ***p<0.001.
